# Maize (*Zea mays L.*) responses to salt stress in terms of root anatomy, respiration and antioxidative enzyme activity

**DOI:** 10.1186/s12870-022-03972-4

**Published:** 2022-12-20

**Authors:** Dandan Hu, Rongfa Li, Shuting Dong, Jiwang Zhang, Bin Zhao, Baizhao Ren, Hao Ren, Haiyan Yao, Ziqiang Wang, Peng Liu

**Affiliations:** 1 College of Agronomy, State Key Laboratory of Crop Biology, Shandong Agricultural University, Tai’an, Shandong 271018 People’s Republic of China; 2 Agricultural Technology Extension Center of Wudi, Binzhou, Shandong 251900 People’s Republic of China; 3 Binzhou Academy of Agricultural Science, Binzhou, Shandong 256603 People’s Republic of China

**Keywords:** Maize, Salt stress, Root anatomy, Root respiration rate, Oxidative injury

## Abstract

**Background:**

Soil salt stress is a problem in the world, which turns into one of the main limiting factors hindering maize production. Salinity significantly affects root physiological processes in maize plants. There are few studies, however, that analyses the response of maize to salt stress in terms of the development of root anatomy and respiration.

**Results:**

We found that the leaf relative water content, photosynthetic characteristics, and catalase activity exhibited a significantly decrease of salt stress treatments. However, salt stress treatments caused the superoxide dismutase activity, peroxidase activity, malondialdehyde content, Na^+^ uptake and translocation rate to be higher than that of control treatments. The detrimental effect of salt stress on YY7 variety was more pronounced than that of JNY658. Under salt stress, the number of root cortical aerenchyma in salt-tolerant JNY658 plants was significantly higher than that of control, as well as a larger cortical cell size and a lower root cortical cell file number, all of which help to maintain higher biomass. The total respiration rate of two varieties exposed to salt stress was lower than that of control treatment, while the alternate oxidative respiration rate was higher, and the root response of JNY658 plants was significant. Under salt stress, the roots net Na^+^ and K^+^ efflux rates of two varieties were higher than those of the control treatment, where the strength of net Na^+^ efflux rate from the roots of JNY658 plants and the net K^+^ efflux rate from roots of YY7 plants was remarkable. The increase in efflux rates reduced the Na^+^ toxicity of the root and helped to maintain its ion balance.

**Conclusion:**

These results demonstrated that salt-tolerant maize varieties incur a relatively low metabolic cost required to establish a higher root cortical aerenchyma, larger cortical cell size and lower root cortical cell file number, significantly reduced the total respiration rate, and that it also increased the alternate oxidative respiration rate, thereby counteracting the detrimental effect of oxidative damage on root respiration of root growth. In addition, Na^+^ uptake on the root surface decreased, the translocation of Na^+^ to the rest of the plant was constrained and the level of Na^+^ accumulation in leaves significantly reduced under salt stress, thus preempting salt-stress induced impediments to the formation of shoot biomass.

## Background

Excessive salinity is a serious environmental stress factor that restricts the productivity and sustainability of agricultural enterprises located in arid and semiarid regions [1–3]. In addition to exacerbating the effects of global warming, the widespread deployment of inappropriate irrigation methods we have witnessed in recent times, especially the excessive use of fertilizers and pesticides, has given rise to a marked worsening of soil salinization around the world [4, 5]. Maize, one of the most important crops in the world, is moderately sensitive to salinity [6]. Like most crops, salinity negatively affects a maize plant’s relative growth rate, osmotic status, transpiration, ion transport, photosynthetic activity, and senescence [7–10]. In addition, an excessive accumulation of salt in a maize plant’s cells can produce reactive oxygen species (ROS), such as hydrogen peroxide, hydroxyl radicals, and superoxide anions, all of which inhibit photosynthetic activity [11, 12]. In order to survive, plants are forced to adapt, and have been observed to respond by deploying various strategies like the extrusion or compartmentalization of toxic ions, the establishment of an enhanced level of biosynthesis of osmolytes, and the activation of ROS scavenging systems [13, 14].

In maize plant cells, salt-induced osmotic effects alter the general metabolic processes and activity levels of enzymes, leading to an excessive accumulation of ROS, which in turn gives rise to oxidative stress [15, 16]. When exposed to oxidative stress, plant cells typically respond by establishing an intricate antioxidant system that regulates redox homeostasis, commonly involving the superoxide dismutase (SOD), catalase (CAT), ascorbate peroxidase (APX) and peroxidase (POD), as well as other free radical scavengers [17, 18]. Zhang et al. [19] showed that in rice roots exposed to saline-alkali stress, the activity of antioxidant enzymes such as SOD, POD, and CAT was significantly elevated, and that this response enhanced the plant’s ability to scavenge ROS, which ultimately mitigated cell damage and improved its chances of survival. In soybean roots, salt stress induces lignification, which is a metabolic process usually accompanied by an increase in POD activity and a significant decrease in H_2_O_2_ content while also is known to be associated with inhibited root growth [20]. When specifically considering salt-tolerant crop genotypes, it has been shown that the SOD and CAT activity are usually depressed, while the APX, guaiacol peroxidase and glutathione reductase activity does not change significantly in the roots of plants subjected to salt stress. In the roots of salt-sensitive genotypes, however, a relatively low enzyme activity of any of these enzymes typically results in an accumulation of ROS, and a subsequent membrane lipid peroxidation that inhibits the growth and development of the root [16, 21].

When a plant is exposed to salt stress during its development, its root system, being the first organ to perceive the stress signal, is incited to adapt its morphological structure to counteract the unfavorable environment it is presented with [22]. One of the most notable adaptations to salt stress observed in the root anatomy of maize plants is RCA formation. RCA formation increases a plant root’s capacity for water and nutrient acquisition, thereby reducing the metabolic costs of soil exploration and allowing it to grow larger [23, 24]. It has also been shown that RCA plays a central role in improving a plant’s capacity to transport oxygen from its shoots to its roots [25]. In addition, substantially elevated RCA and CCS have been observed in barley, wheat and rice exposed to water stress, which was shown to be the result of the development of an elevated number of xylem vessels [26, 27]. Water stress also negatively affects the RCA in the root hairs of maize, but it improves the RCA of mature roots [28]. Zhu et al. [29] demonstrated that the formation of RCA enhances the drought tolerance of maize, mainly because the adapted root architecture provides more airspace, which reduces the level of root respiration.

In addition to these morphological adaptions, plants also develop biochemical responses to salt stress. Mitochondrial electron transport pathways in plants are mainly comprised of cytochrome oxidase (COX) respiratory pathways and alternate oxidase (AOX) respiratory pathways, both of which are susceptible to environmental stress [30]. In many plants, environmental and chemical stress stimulates AOX synthesis, while in the absence of stress it generally remains at a low level [31]. Several studies have shown that AOX plays an important role in plant adaptations to environmental stresses such as excessive salinity, cold, waterlogging, drought and high light [32–36]. The salt tolerance of plants is associated with elevated AOX gene expression and increased enzyme activity [34, 37]. AOX is insensitive to cyanide, but its activity can be inhibited by salicylhydroxamic acid (SHAM) [38]. In cases where the COX respiratory pathway is compromised due to environmental stress, the AOX respiratory pathway can uphold respiration by directly receiving electrons from ubiquitin, which reduces oxygen to water. In addition, AOX consumes excessive reduction equivalents produced by chloroplasts, thus maintaining intracellular redox homeostasis [39–41]. Studies showed that both the total respiration rate and the AOX respiration rate of leaves decreased under salt stress [34], confirming that AOX plays a critical role in maintaining respiration and prevents extensive oxidative damage and functional loss of mitochondria and chloroplasts under conditions of water stress [41]. It has also been observed that the cells of leaves from alfalfa plants exposed to severe short-term salt stress could improve their photosynthetic rate and water content by deploying a combination of alternate oxidative respiration and organic acid and amino acid metabolic processes, thus improving the alfalfa’s salt tolerance [42]. The findings from these studies confirm that in order to develop agricultural practices aimed at improving the salt tolerance of crop plants, it is necessary to understand the related physiological and biochemical processes in maize plants.

In order to cope with salt stress, it is essential to maintain ion homeostasis in plants. Under salt stress, some studies showed a sharp increase in the Na^+^ level and a decrease in the K^+^ level in roots [43, 44]. In salt-tolerant genotypes, lower Na^+^/K^+^ ratios enable plants to grow well under saline environments and to preserve cellular metabolism by promoting protein synthesis, regulating enzyme activation, photosynthesis, osmoregulation and maintaining cell turgor [45]. Salt-induced phytotoxicity increased the maize plant tissue concentration of Na^+^/K^+^ ionic ratio, Na^+^ translocation (root to shoot), and its uptake [16]. Salt stress increased the translocation factor of Na^+^ and K^+^ and reduced the selective transport of roots for K^+^ over Na^+^ in rice [46].

The study presented in this paper had the following objectives: (1) evaluate the effects of salt stress on the antioxidative enzyme activity and the level of lipid peroxidation in the leaves and roots of two maize genotypes with different salt tolerances; (2) compare the differences in and analyze the relationship between the root anatomy traits and respiration rates of maize plants subjected to salt stress and maize plants grown under standard conditions; and (3) explore how exposure to salt stress affects a maize root’s uptake and transport of Na^+^ and K^+^.

## Results

### Salt stress treatment affects plant growth and the leaf relative water content (RWC)

**Fig. 1:**
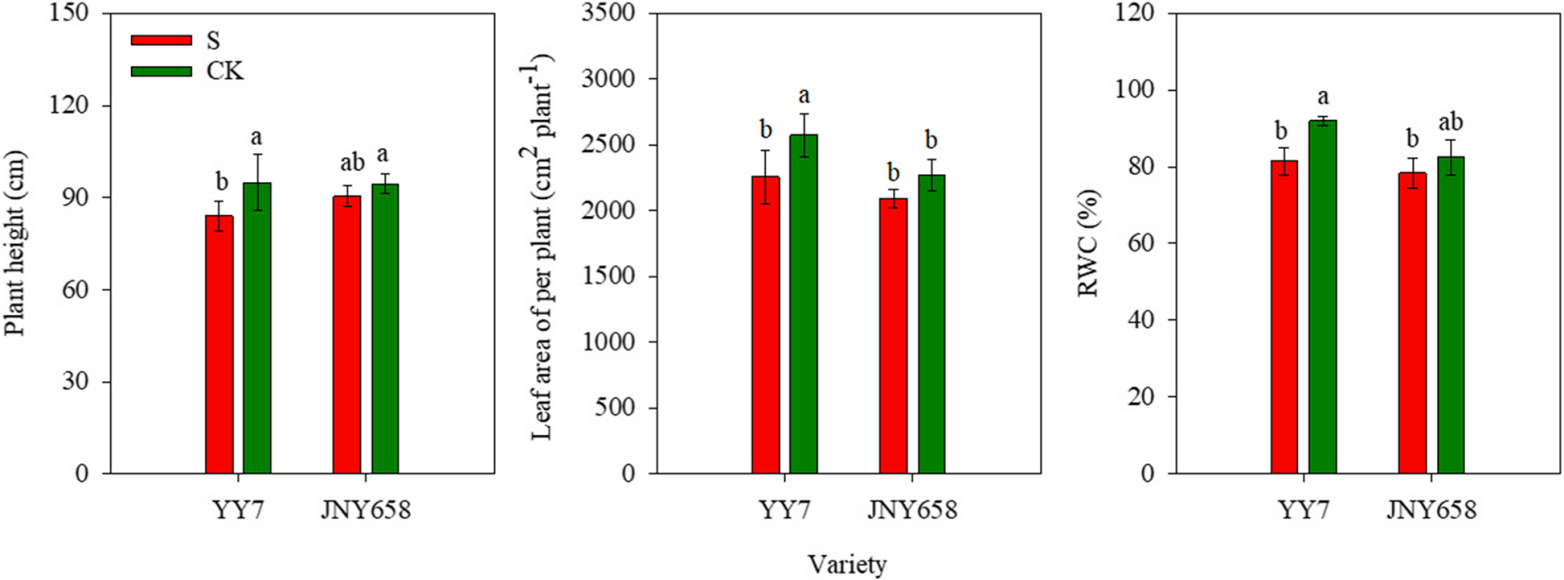
Effect of salt stress on plant height, leaf area and RWC of salt-tolerant (JNY658) and salt-sensitive (YY7) maize varieties. Bars being annotated with different lowercase letters indicates they are significantly different at *P* < 0.05

The two-way ANOVA showed that both the salinity and the choice of maize variety significantly affected the RWC (*P* < 0.05; Table 1). At V6 stage, the height and leaf area of YY7 plants in salt stress (S) treatment were markedly lower than those of plants from the CK treatment. Salt stress also significantly reduced the height of JNY658 plants, but not their leaf area. YY7 plants subjected to the S treatment were 11.37% shorter than plants from the CK group, and their leaf area was 12.32% smaller. At 11.44% less, the difference in RWC between plants from the S and CK groups was most pronounced for the YY7 variety. For JNY658 plants, we found a much smaller difference of only 4.95% (Fig. 1).

**Table 1:** Summary of the ANOVA analysis of the physiological parameters of the leaves of plants subjected to salt stress, and statistical differences between the result from the soil and hydroponics culture experiments

Effect	RWC	Pn	SOD	POD	CAT	MDA	Leaf Na^+^ content	SD
Salt	7.94^*^	7.59^*^	43.61^***^	110.22^***^	10.03^*^	9.27^*^	99.04^***^	22.73^***^
Variety	5.87^*^	0.27^ns^	210.64^***^	4.00^ns^	25.00^**^	102.65^***^	3.39^ns^	0.95^ns^
Salt ×Variety	1.54^ns^	0.27^ns^	0.57^ns^	0.09^ns^	1.78^ns^	0.49^*^	4.47^*^	7.33^*^

*Note*: ^⁎^Significant at *P* < 0.05; ^⁎⁎^Significant at *P* < 0.01; ^***^Significant at *P* < 0.001; ^ns^ non-significant

### Salt stress treatment affects photosynthesis parameters

**Fig. 2:**
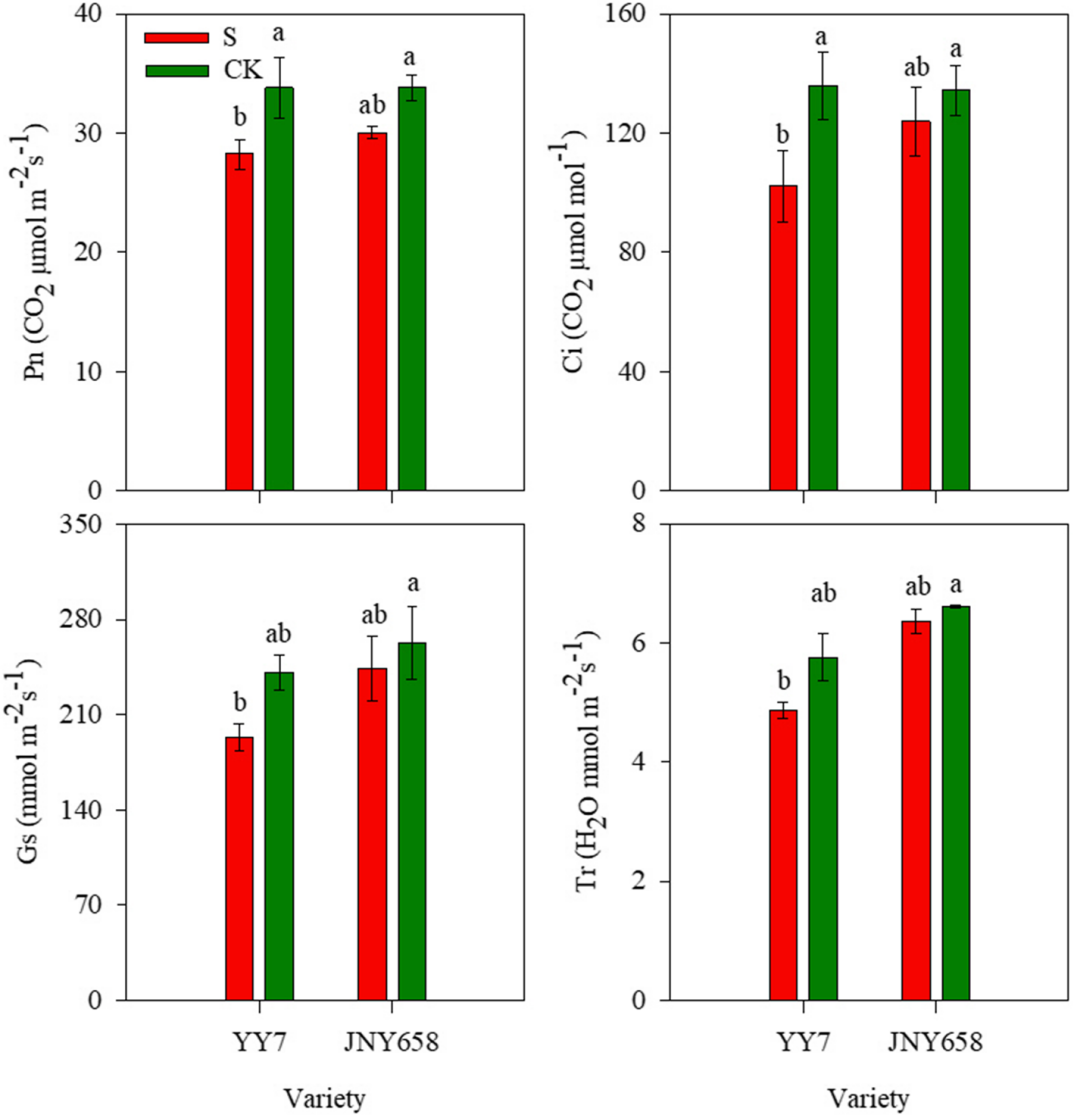
Effect of salt stress on the photosynthetic characteristics of salt-tolerant (JNY658) and salt-sensitive (YY7) maize varieties. Bars being annotated with different lowercase letters indicates they are significantly different at *P* < 0.05

Salt stress had a significant effect on the net photosynthetic rate in leaves (*P* < 0.05; Table 1). Our results showed that the net photosynthetic rate (Pn), intercellular CO_2_ concentration (Ci), stomatal conductance (Gs) and transpiration rate (Tr) in the leaves of plants subjected to the S treatment were all lower than the corresponding values in those of the CK treatment. For JNY658 plants, there was no significant difference between the Pn and the Ci of plants subjected to the S treatment and plants from the CK treatment, but the Pn and Ci of YY7 plants subjected to the S treatment were a significant 19.69 and 24.76% lower than those of plants from the CK treatment respectively (Fig. 2).

### Salt stress treatment affects root respiration

**Fig. 3:**
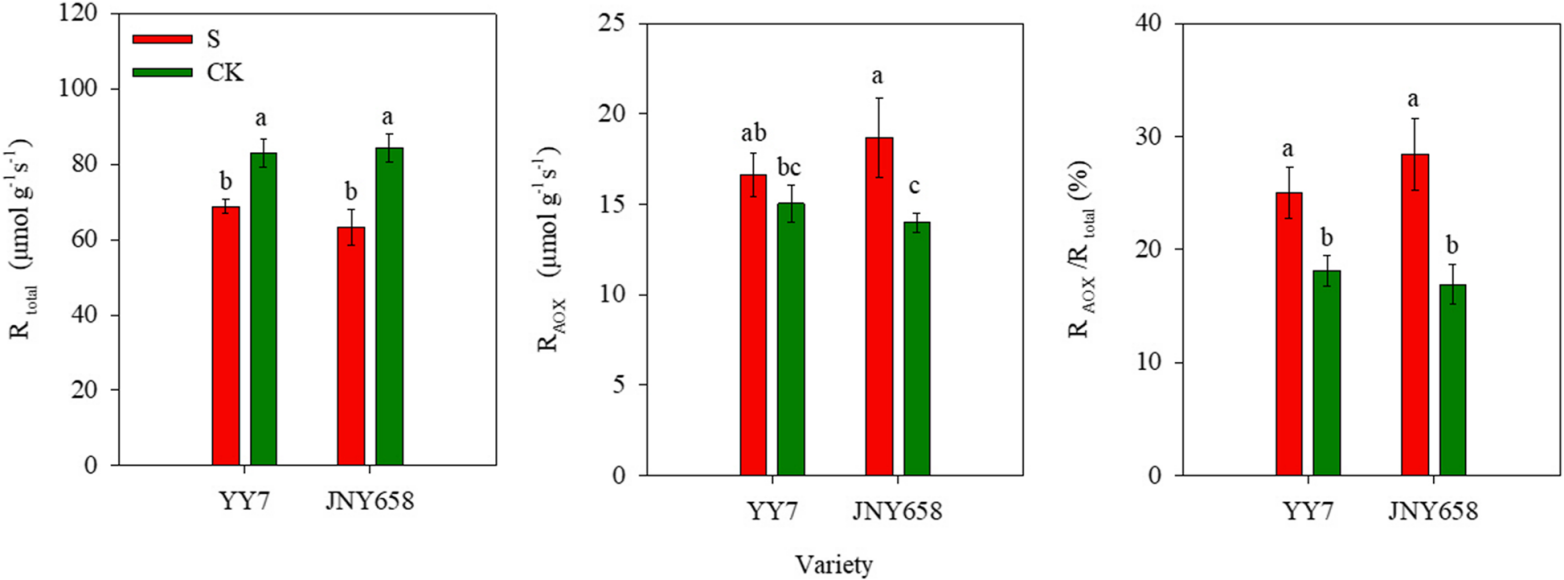
Effect of salt stress on the respiration rates in the root systems of salt-tolerant (JNY658) and salt-sensitive (YY7) maize varieties. Bars being annotated with different lowercase letters indicates they are significantly different at *P* < 0.05

Our results showed that exposure to salt stress affected both the total respiration rate (R_Total_) and the alternate oxidative respiration rate (R_AOX_) of maize plants (Table 2). The R_Total_ of roots of plants of both varieties included in our experiment was lower than that of the roots of plants from the respective CK treatments, while the R_AOX_ and R_AOX_/R_Total_ were higher. The R_Total_ of the roots from YY7 and JNY658 plants subjected to the S treatment was 17.08 and 25.05% lower than that of the corresponding CK treatment, respectively; the R_AOX_ was 10.43% and 33.64 higher; and the R_AOX_/R_Total_ was 38.36 and 67.93% higher (Fig. 3), confirming that JNY958 responded more strongly to salt stress than YY7 plants did.

**Table 2:** Summary of the ANOVA analysis of the physiological parameters of the roots of plants subjected to salt stress, and statistical differences between the result from the soil and hydroponics culture experiments

Effect	SOD	POD	CAT	MDA	RCA	R_Total_	R_AOX_	Na^+^ translocation	Na^+^ uptake	RD
Salt	138.50^***^	11.82^**^	99.49^***^	34.41^***^	71.87^***^	30.56^***^	29.44^***^	14.74^**^	204.84^***^	23.32^***^
Variety	52.38^***^	18.28^**^	5.40^*^	34.96^***^	8.39^*^	1.25^ns^	0.23^ns^	0.001^ns^	27.13^***^	3.97^ns^
Salt ×Variety	5.08^*^	2.39^ns^	5.40^*^	5.69^*^	1.90^ns^	0.12^ns^	7.00^*^	0.59^ns^	20.91^***^	7.14^*^

*Note*: ^⁎^Significant at *P* < 0.05; ^⁎⁎^Significant at *P* < 0.01; ^***^Significant at *P* < 0.001; ^ns^ non-significant

### Salt stress treatment affects root anatomical traits

**Fig. 4:**
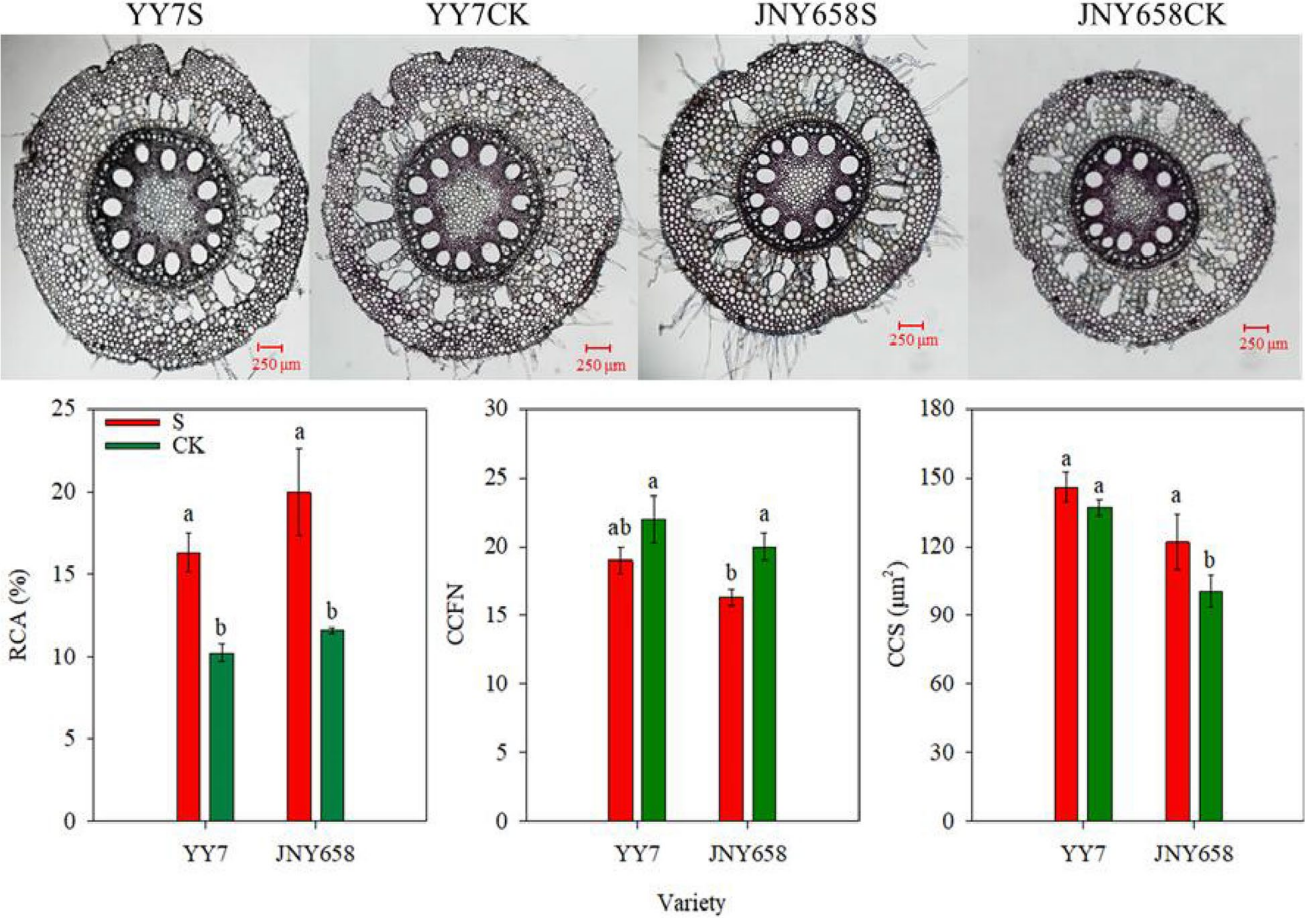
Effect of salt stress on the anatomic traits of salt-tolerant (JNY658) and salt-sensitive (YY7) maize varieties. Bars being annotated with different lowercase letters indicates they are significantly different at *P* < 0.05. RCA: root cortical aerenchyma; CCS: cortical cell size; CCFN: cortical cell file number

A two-way ANOVA showed that both exposure to salt stress and the salt-tolerance of the used maize variety significantly affected the root cortical aerenchyma (RCA) (*P* < 0.05; Table 2). We found that for both varieties included in our experiment, the RCA and cortical cell size (CCS) of plants subjected to the S treatment were higher than those of plants subjected to the CK treatment. In contrast, the cortical cell file number (CCFN) was smaller. The RCA of YY7 seedlings subjected to the S treatment was 58.49% higher than that of seedlings subjected to the CK treatment. And their CCS was 6.51% higher and their CCFN was significantly 13.64% lower. The response of JNY658 plants was even stronger: the RCA of JNY658 plants subjected to the S treatment was 72.14% higher than those from the CK treatment, their CCS was 21.30% higher and their CCFN was 18.33% lower (Fig. 4).

### Salt stress treatment affects antioxidant enzyme activity

To evaluate the role of the antioxidant system in a maize plant’s response to salt stress, we analyzed the activity of antioxidant enzymes in its leaves and roots. We found that both exposure to salt stress and the plant’s variety, as well as the interaction between those factors, significantly affected the SOD and CAT activity in leaves and roots (*P* < 0.05; Tables1 and 2). The SOD and POD activities in the leaves and roots of plants of both varieties subjected to salt stress were higher than that in the leaves and roots of plants from the control treatment. The response exhibited by JNY658 plants, however, was more pronounced than that exhibited by YY7 plants. The SOD activity in the leaves of YY7 plants that had been subjected to the S treatment was a significant 16.87% higher than that in leaves of plants from the CK treatment and in root it was 28.58% higher. JNY658 plants responded even more strongly: the SOD activity in their leaves was as much as 34.96% higher and that in their roots was 55.40% higher than the corresponding values in leaves and roots of plants from the control treatment. The POD activity exhibited as a similar trend, except for the POD activity in the roots from YY7 pants, which was not significantly different between the two treatments.

**Fig. 5:**
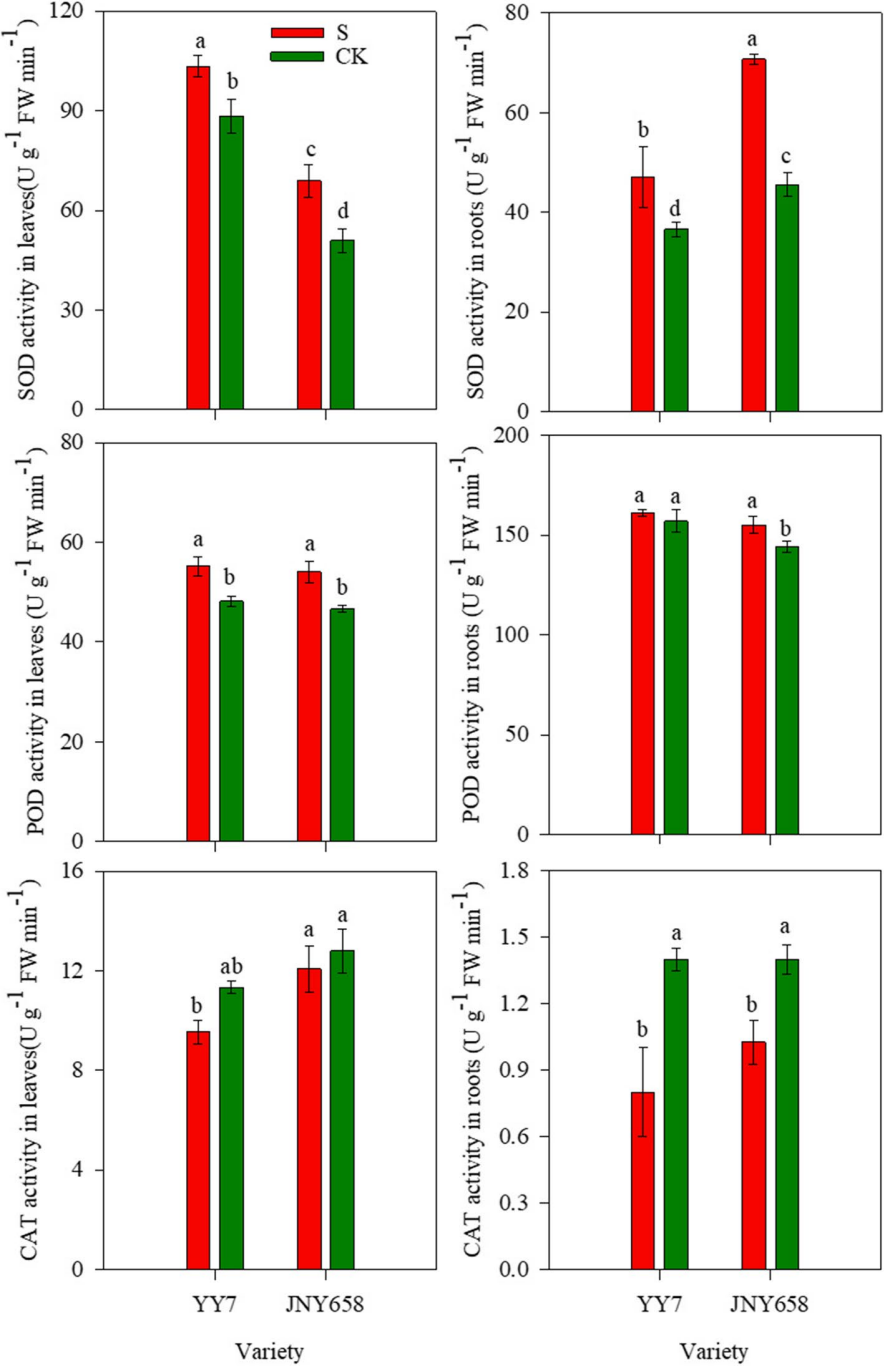
Effect of salt stress on the antioxidative system of salt-tolerant (JNY658) and salt-sensitive (YY7) maize varieties. Bars being annotated with different lowercase letters indicates they are significantly different at *P* < 0.05

The CAT activity in the leaves and roots of seedlings subjected to the S treatment was lower than that in the leaves and roots of seedlings subjected to the CK treatment. The CAT activity in the leaves of YY7 plants subjected to the S treatment was a significant 15.88% lower than that in the leaves of plants subjected to the CK treatment, and in root it was 42.86% lower. The corresponding values in the leaves and roots of JNY658 plants subjected to the S treatment were 5.73 and 26.67% lower than those in the leaves and roots of plants from the control treatment, respectively (Fig. 5).

### Salt stress treatment affects MDA content

**Fig. 6:**
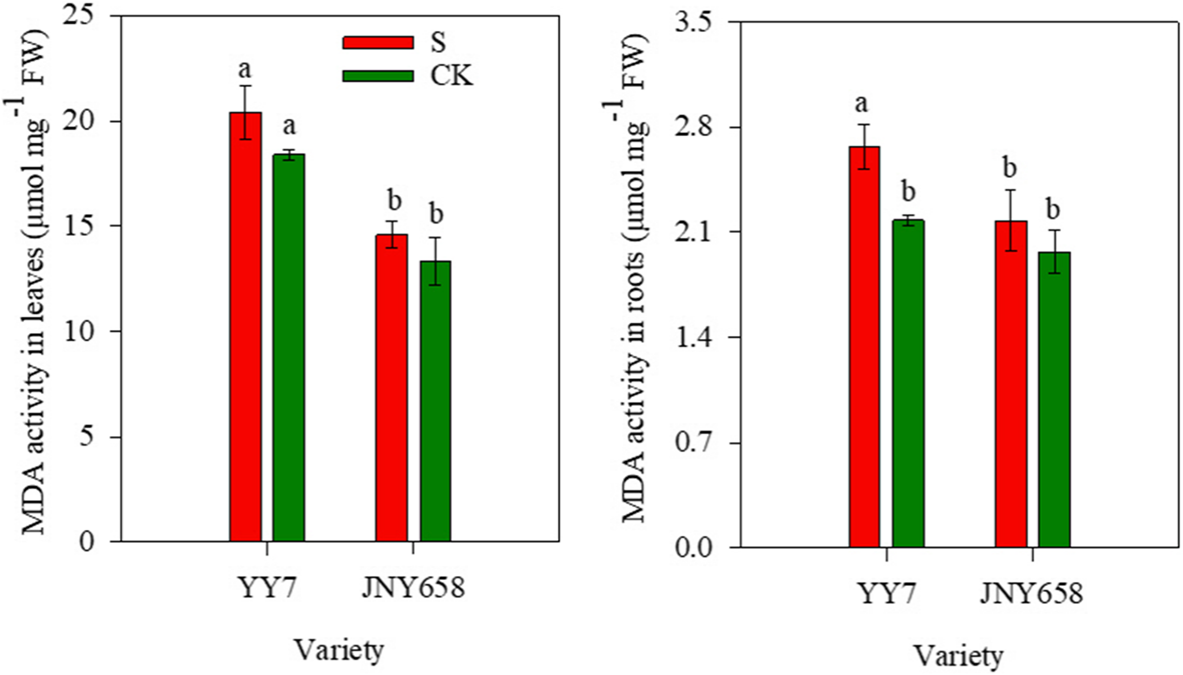
Effect of salt stress on the MDA and proline content of salt-tolerant (JNY658) and salt-sensitive (YY7) maize varieties. Bars being annotated with different lowercase letters indicates they are significantly different at *P* < 0.05

Salt stress, variety, and their interaction significantly (*P* < 0.05) affected the MDA content in the leaves and roots (Tables 1 and 2). the MDA content in the leaves and roots of plants of both varieties subjected to the S treatment was higher than that in the leaves and roots of plants subjected to the CK treatment; the only significant difference (22.46%, *P* < 0.05) was observed in the roots from YY7 plants (Fig. 6).

### Salt stress treatment affects net Na^+^ and K^+^ flux

The net Na^+^ and K^+^ flux (defined as the difference between efflux and influx) from the roots of both JNY658 and YY7 plants subjected to the S treatment was markedly higher than that from the roots of plants subjected to the CK treatments, but JNY658 plants responded significantly stronger to salt stress than YY7 plants: The average net Na^+^ efflux from the roots of JNY658 plants subjected to the S treatment was 2.43 times higher than that from the roots of plants subjected to the CK treatment, while for YY7 plants it was only 1.67 times higher.

**Fig. 7:**
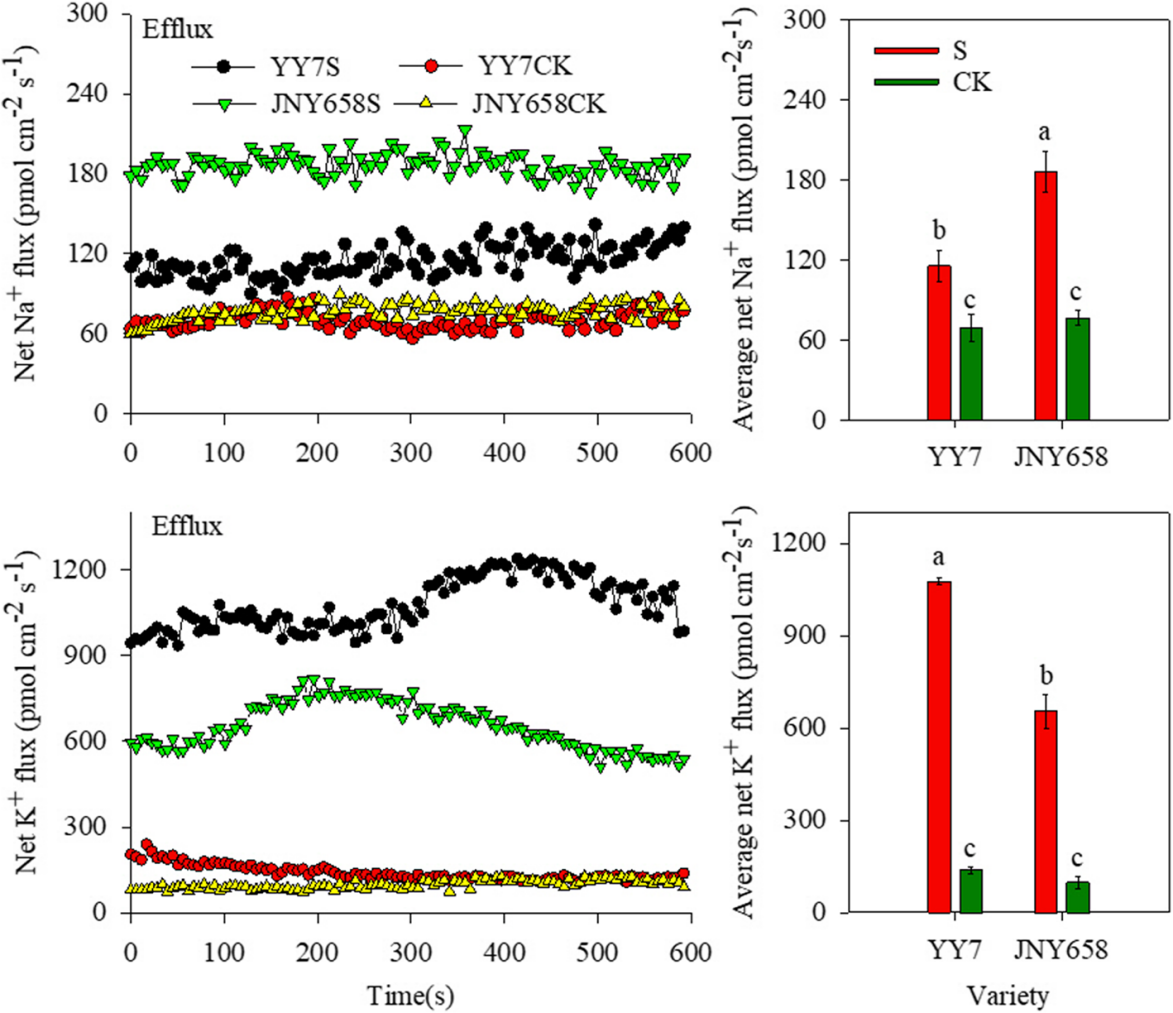
Effect of salt stress on the net Na^+^ and K^+^ flux in the roots of salt-tolerant (JNY658) and salt-sensitive (YY7) maize varieties. Bars being annotated with different lowercase letters indicates they are significantly different at *P* < 0.05

Although the net K^+^ flux from the roots of plants of both varieties subjected to the CK treatment were not significantly different, we found that in plants subjected to the S treatment, it was markedly higher for the YY7 variety than for the JNY658 variety: The average net K^+^ flux from the roots of YY7 plants exposed to salt stress was 7.77 times higher than that from the roots of plants from the control treatment, while for the roots of JNY658 plants it was only 6.68 times higher (Fig. 7).

### Salt stress treatment affects root Na^+^ translocation, root Na^+^ uptake and leaf Na^+^ content

**Fig. 8:**
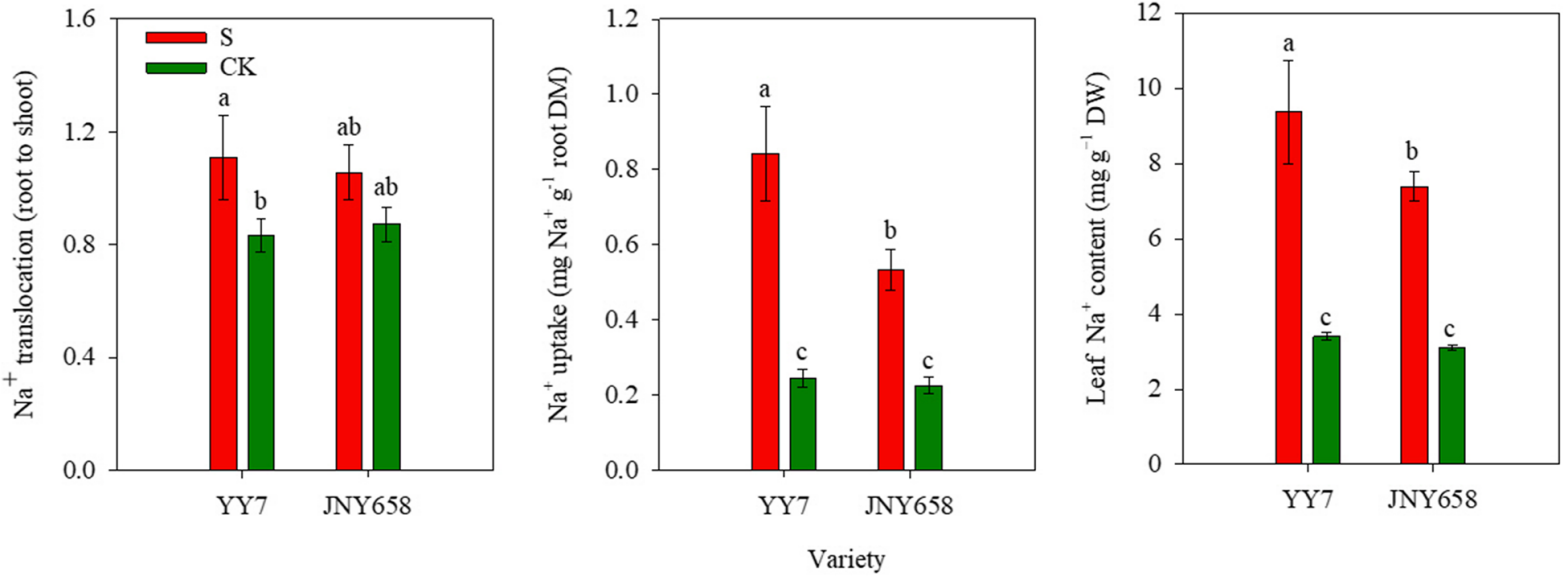
Effect of salt stress on the Na^+^ translocation, the Na^+^ uptake at the root surface and the Na^+^ content in leaves of salt-tolerant (JNY658) and salt-sensitive (YY7) maize varieties. Bars being annotated with different lowercase letters indicates they are significantly different at *P* < 0.05

Exposure to salt stress significantly affected the root Na^+^ translocation, the root Na^+^ uptake and the leaf Na^+^ content of maize plants (*P* < 0.05; Tables 1 and 2). In terms of Na^+^ translocation and Na^+^ uptake, YY7 plants responded significantly more stronger to salt stress than JNY658 plants did. The level of Na^+^ translocation from the roots to the shoots of YY7 seedlings subjected to the S treatment was 32.93% higher than that from the roots to the shoots of seedlings from the control treatment, while we observed no significant difference in JNY658 seedlings. The Na^+^ net uptake at the root surface of YY7 seedlings subjected to salt stress was 3.43 times higher than that at the root surface of seedlings from the control treatment, while for JNY658 seedlings the corresponding value was only 2.37 times higher. The Na^+^ content of the leaves of YY7 plants subjected to the S treatment was 175.26% higher than that of the leaves of plants subjected to the CK treatment and for JNY659 plants it was 139.23% higher. (Fig. 8).

### Salt stress treatment affects dry matter weight and STI

**Fig. 9:**
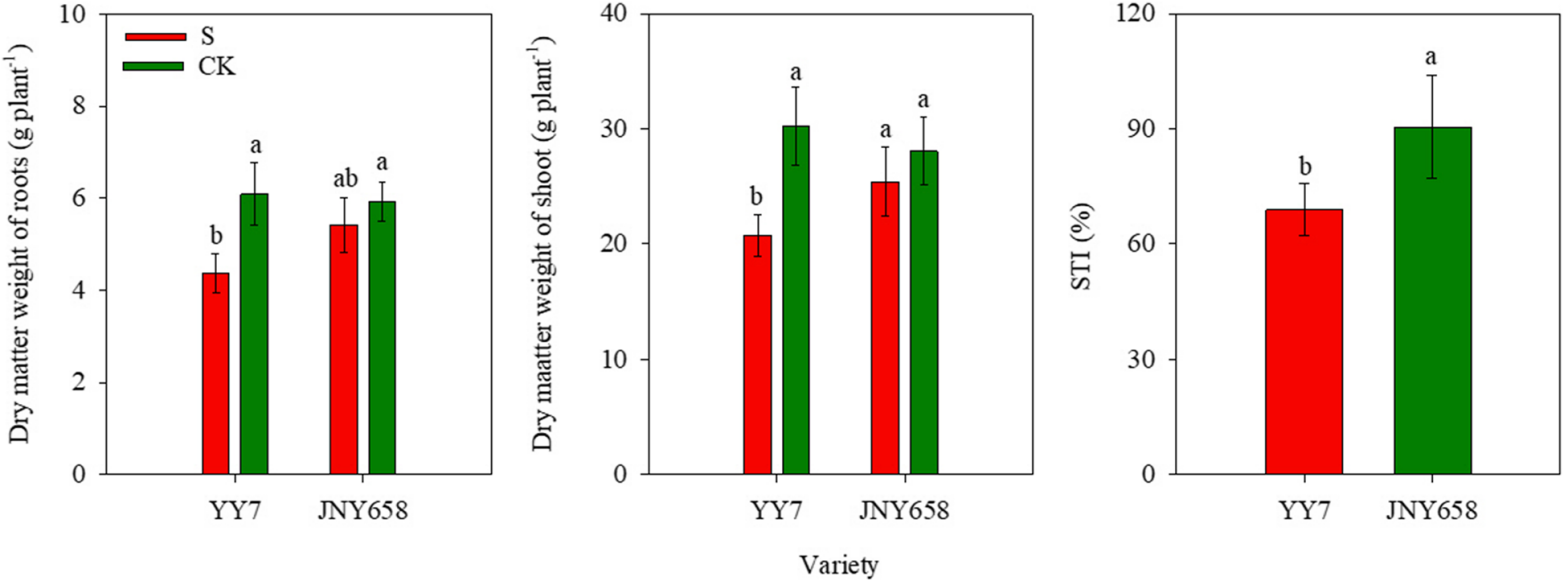
Effect of salt stress on the dry matter weight and the salt tolerance index (STI) of salt-tolerant (JNY658) and salt sensitive (YY7) maize varieties. Bars being annotated with different lowercase letters indicates they are significantly different at *P* < 0.05

Both exposure to salt stress and the interaction between salinity and the used maize variety significantly affected the shoot and root biomass of YY7 plants, while the effect on the biomass of JNY658 plants was not significant (*P* < 0.05; Tables 1 and 2). The biomass contained in the roots and shoots of harvested YY7 plants subjected to the S treatment were 39.17 and 45.95% lower than that of plants subjected to the CK treatment, respectively, and the corresponding values for the roots and shoots of JNY658 plants were 10.33 and 9.06% lower. The STI of JNY658 plants subjected to the S treatment was 31.38% higher than that of YY7 plants subjected to the same treatment (Fig. 9).

### Correlation analysis

The leaf Na^+^ content exhibited a significant positive correlation with the Gs, the leaf SOD activity, the leaf POD activity, the leaf MDA content, the RCA, the CCS, R_AOX_, the root SOD, the root MDA and the Na^+^ uptake, as well as a significant negative correlation with the RWC, the Pn, the Ci, the leaf CAT activity, the CCFN, the R_Total_ and the root CAT activity. The RD exhibited a significant positive correlation with the RWC, the Pn, the Ci, the leaf CAT content, the CCFN, the R_Total_ and the root CAT content, as well as a significant negative correlation with the Gs, the leaf SOD activity, the leaf POD activity, the leaf MDA content, the RCA, the CCS, the root SOD activity, the root MDA content, the Na^+^ uptake, and the leaf Na^+^ content. The SD exhibited a significant positive correlation with the RWC, the Pn, the Ci, the leaf CAT content, the CCFN, the R_Total_ and the root CAT content, and RD, SD had a significant negative correlation with the leaf SOD activity, the leaf POD activity, the leaf MDA content, the RCA, the CCS, the R_AOX_, the root SOD activity, the root MDA content, the Na^+^ uptake, and the leaf Na^+^ content.

## Discussion

### Comparison of the responses of maize varieties with different salt tolerances to salt stress in terms of their photosynthetic parameters

The accumulation of Na^+^ in plants drastically affects most of their physiological attributes, including the photosynthetic rate, the transpiration rate, and stomatal opening and closing [47]. Jiang et al. [48] suggested that a higher tolerance to salt stress, which mitigates the negative impact of salt stress on a plant’s photosynthetic machinery due to effects like Na^+^ accumulation, may improve a plant’s photosynthetic characteristics. Exposure to salt stress gives rise to significantly reduced values of the Pn, Gs, Ci, and Tr in tomato, peanut and cotton plants [49–51]. Salt stress also engenders a smaller stomatal aperture, a lower intercellular CO_2_ concentration, and the impairment of photosynthetic electron transport, which results in the production of ROS [10, 12]. The study presented in this paper reveals that the leaves of maize plants that had been subjected to a treatment involving the application of a high amount of NaCl exhibited a lower Pn, Gs, and Ci than the leaves of plants from the control treatment and that the salt-sensitive variety YY7 exhibited a stronger response than the salt-tolerant variety JNY658 (Fig. 2). The difference in response might be due to the fact that plants of the YY7 variety exposed to salt stress suffer from a markedly reduced RWC, which results in reduced water uptake and disrupts the plant’s water balance (Fig. 1) [16]. In turn, the relatively low RWC of salt-sensitive maize cultivars grown in a saline environment could be caused by a low cell turgidity or a lack of capacity to transport water from the roots to the shoots [52, 53].

### Comparison of the responses of maize varieties with different salt tolerances to salt stress in terms of their root anatomical traits and respiration rates

**Fig. 10:**
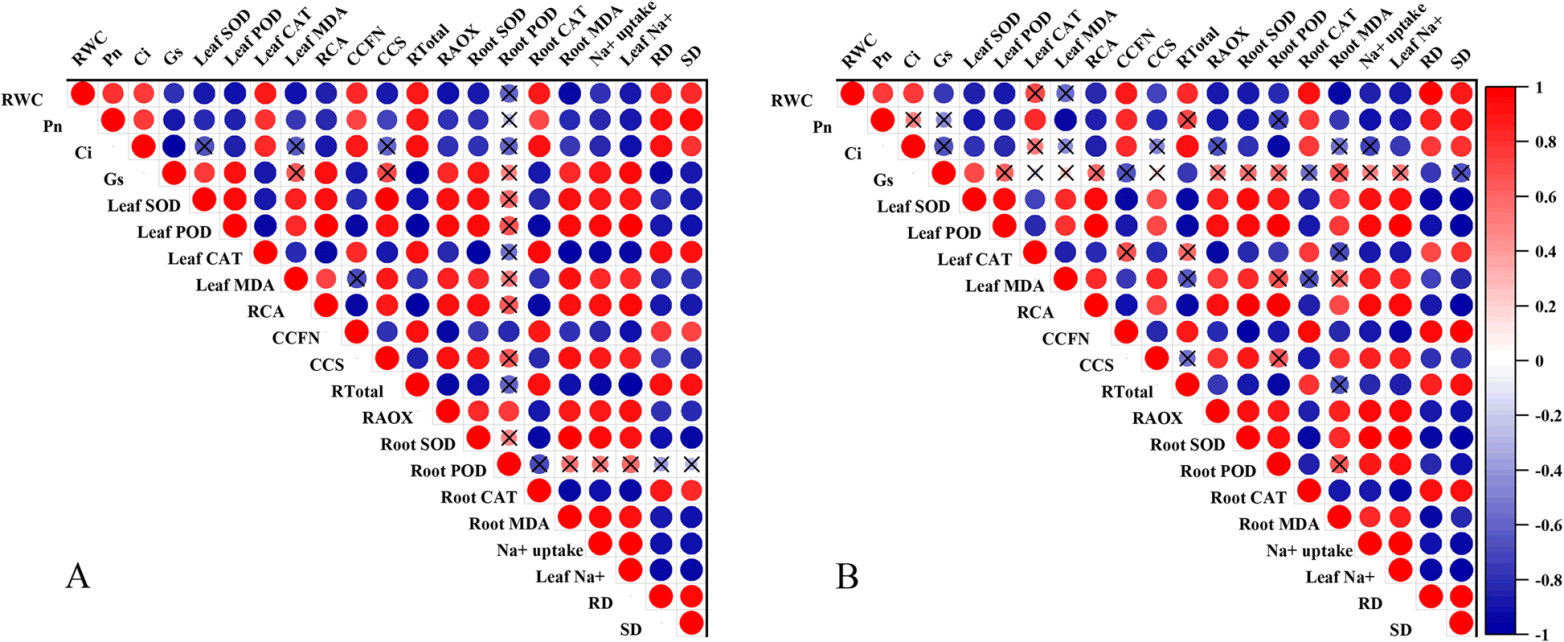
Pearson correlation analysis between various measured attributes of JNY658 (**A**) and YY7 (**B**) plants (*P* < 0.05). The abbreviations are as following: RWC: the leaf relative water content; POD: peroxidase activity; SOD: superoxidase activity; CAT: catalase activity; MDA: malondialdehyde contents; Pn: leaf net photosynthetic; Tr: transpiration rate; Gs: stomatal conductance; Ci: internal CO_2_ concentration; SD: shoot dry weight; RD: root dry weight

Exposing plants to salt stress during the formation of the RCA inhibits the development of the active cortex area, resulting in a reduced respiration rate and a lower nutrient content of the root tissue, which prompts the root system to continue the process of soil exploration [54]. The development of RCA, which enlarges the area of the channels in the root cortex, is considered to play an important role in reducing the metabolic cost of soil exploration and improving a root’s capacity to take up water [55, 56]. The mechanism by which RCA is formed in maize plants involves programmed cell death [57], which reduces the root’s nutrient content and respiration rate [58]. When the availability of water and nutrients in the soil is limited, the formation of RCA improves the capacity of the root system to absorb water and nutrients and supply them to the rest of the plant [56, 59]. Zhu et al. [29] found that under drought conditions maize genotypes with a high RCA had a 5-fold higher biomass and an 8-fold higher yield than low RCA genotypes. Water stress is known to drastically increase RCA formation [60]. In addition, Chimungu et al. [61] have shown that plants of varieties with a relatively low CCFN were able to maintain a greater RWC and Gs when exposed to water stress. Several studies have suggested that the high RCA, large CCS, and low CCFN induced by water stress reduced a plant’s root respiration, increased its rooting depth, and enhanced its capacity to acquire water [56, 61, 62]. The results of our study showed that, with respect to developing a higher RCA and CCS and a lower CCFN, JNY658 seedlings responded significantly stronger to salt stress than YY7 seedlings did (Fig. 6). It should also be noted that the RCA and CCS exhibited a significant negative correlation with the R_Total_, whereas for the CCFN the same correlation was significantly positive (Fig. 10). These findings confirm that the way JNY658 plants respond to salt stress, which is to say by developing a higher RCA, a larger CCS, and a reduced CCFN, reduces the respiration rate and maintain the water and nutrient uptake capacity of their roots [63]. In addition, our results suggest that the relatively low CCFN of JNY658 plants subjected to salt stress helped them to maintain a higher RWC and Gs, which benefits overall plant growth and biomass formation (Figs. 1, 2, and 9).

Responding to water stress by reducing the level of root respiration can help a plant to reduce the metabolic rate associated with soil exploration by the root system and improve its capacity for nutrient and water absorption, thus increasing crop yield [54, 56]. Several studies have provided evidence that AOX, in addition to playing a general role in maintaining cellular energy homeostasis, may be involved in processes that limit the formation of mitochondrial ROS in tobacco, watermelon, and alfalfa plants exposed to environmental stress [36, 64, 65], suggesting that it might play an essential role in stress responses [66]. When faced with insufficient P availability, tobacco roots have been shown to respond by increasing their AOX respiration rate, thereby increasing its proportion to the total respiration rate [67]. In light of its capacity to influence antioxidant enzyme activity, it has also been suggested that AOX might be involved in responses to low-temperature stress such as those endowing sweet potatoes and chickpeas with a high level of cold tolerance [35, 68]. It has been shown that tomato plants subjected to salt stress significantly decreased their R_Total_ while significantly increasing their R_AOX_, thus enlarging R_AOX_/R_Total_ and improving their salt tolerance [34]. In addition, AOX overexpression in response to extreme drought has been shown to reduce oxidative damage, while AOX knockdown promotes oxidative damage [41]. Our findings indicate that the formation of more RCA caused the respiration rate of the roots of plants of both varieties subjected to salt stress to be lower than that of the roots of plants from the control treatment. The R_AOX_ and R_AOX_/R_Total_ of the roots of JNY658 plants subjected to salt stress showed were significantly higher than those of the roots of plants from the control treatment, while the roots of YY7 plants exhibited no significant differences (Fig. 8). JNY658 plants mitigate the effects induced by salt toxicity by changing the root respiratory chain transmission pathway, and they also reduce the root’s metabolic costs through establishing a more efficient respiration process, which helps to maintain the plant’s growth and development. In addition, R_AOX_ exhibited a significant positive correlation with the SOD and POD activity in roots, but it negatively correlated with the MDA content of roots (Fig. 10). In terms of the increase in SOD and POD activities in their roots, the response of JNY658 plants subjected to salt stress was significantly stronger than that of YY7 plants, but in terms of the increase in MDA content it was the other way round. This suggests that exposure to salt stress significantly increased the R_AOX_/R_Total_ of the roots of JNY658 plants, which served to regulate the antioxidant enzyme activity and lipid peroxidation, thus mitigating oxidative damage and improving salt tolerance.

### Comparison of the responses of maize varieties with different salt tolerances to salt stress in terms of their peroxide scavenging capacity

Antioxidant responses play an important role in improving a plant’s tolerance to salt stress [69]. When the ROS scavenging ability of plants reaches a certain limit, excess ROS can damage cells and cause oxidative stress. In order to regulate redox homeostasis, plants have developed complex antioxidant responses to environmental stress, including the deployment of enzymes that scavenge free radicals, such as SOD, POD, CAT and APX [17, 70]. SOD mainly scavenges O_2_^−^, and CAT and POD mainly scavenge the highly toxic substance H_2_O_2_ by decomposing it into water and oxygen [69, 71]. Previous studies have shown that exposure to salt stress significantly increased the SOD, POD and CAT activity in and the MDA content of wheat, peanut, tomato, cucumber, and Arabidopsis plants [48, 50, 72–75]. Another study demonstrated that treatments involving salt stress decreased the CAT activity in tomato seedlings [76, 77]. Furthermore, salt tolerance has been attributed to enhanced APX, CAT and SOD activity in rice seedlings [78]. Sheikh-Mohamadi et al. [79] demonstrated that a plant’s higher tolerance to salt is associated with a lower MDA content and that its MDA content correlates negatively with its salt resistance. In our study, we found that after subjecting them to salt stress, the SOD and POD activity and the MDA content of the roots and leaves of plants of both maize varieties increased, while the CAT activity was lower [80]. In terms of the changes in MDA content, the response of the salt-sensitive YY7 variety was significantly stronger than that of the JNY658 variety (Figs. 5 and 6), while in terms of the changes in SOD and POD activity and MDA content it was the other way round. Thus, we were able to conclude that oxidative stress plays an important role in maize plants exposed to salt stress and that the response mechanisms protecting the leaves and roots of JNY658 plants against salt stress-induced oxidative damage involved the maintenance and enhancement of the activity of antioxidant enzymes that help to mitigate NaCl-induced oxidative damage and maintain a higher biomass formation.

### Comparison of the responses of maize varieties with different salt tolerances to salt stress in terms of their ability to maintain the K^+^ and Na^+^ ratio in their roots

The damage to plant cells and the resultant growth defects observed in crops exposed to salt stress are primarily caused by the excessive uptake and accumulation of Na^+^ and Cl^−^ ions [81]. Because K^+^ is required by the normal metabolism of plants, it is very important that any mechanisms contributing to the salt tolerance of plants maintain the K^+^ content of their leaves and roots [82]. Salt stress has been shown to compromise plant metabolism by inducing K^+^/Na^+^ imbalances [83], which led to significant Na^+^ and K^+^ efflux from the meristematic and elongation zones of rice roots [46]. NMT is a practically useful technology for non-invasively investigating the ion flux in plant roots. In our study, we analyzed the Na^+^ and K^+^ efflux characteristics of the roots of both maize varieties included in our experiment after having been subjected to a treatment involving salt stress. In the roots of plants of the salt-tolerant maize variety JNY658, we observed a strong Na^+^ efflux, which may be due to the fact that SOS is mainly expressed in roots [63]. We also found that the K^+^ efflux from the roots of JNY658 plants was weaker than that from the roots of YY7 plants (Fig. 7). This finding indicates that the main reason why JNY658 has a higher salt tolerance than YY7 is its capability to maintain a lower Na^+^/K^+^ ratio in its roots. Yan et al. [84] reported that salt sensitive honeysuckle varieties exposed to salt stress tend to accumulate Na^+^ in their leaves, leading them to exhibit severe toxicity symptoms. In our study, we found that the Na^+^ translocation from root to shoot in YY7 subjected to salt stress was significantly higher than that in plants from the control treatment, but we found no significant response in JNY658 plants, which suggests that salt-tolerant maize varieties have the capacity to inhibit the Na^+^ transport to their leaves when exposed to salt stress. Accordingly, our results revealed that the Na^+^ concentration in the lower leaves of JNY658 plants depends on a higher Na^+^ efflux from the root, as well as a restriction of the amount of Na^+^ transported from roots to leaves (Fig. 8).

## Conclusions

In conclusion, our study shows that, compared with the salt-sensitive variety YY7, the salt-tolerant maize variety JNY658 responded to salt stress by establishing a higher RCA and CCS, and a lower CCFN in its roots, as well as by reducing the R_Total_ and enhancing the R_AOX_, thus mitigating the amount of oxidative damage to cells. In addition, salt-tolerant maize variety had the capacity to lower the ionic toxicity in its leaves by maintaining a greater root Na^+^ extrusion, thereby restricting the amount of Na^+^ transported to its leaves and establishing a stronger root Na^+^ efflux. The combined effect from these responses might be responsible for the capacity of JNY658 plants to maintain a relatively high photosynthetic activity in their leaves when exposed to salt stress.

## Methods

### Experimental subjects

After evaluating the data on a total of 71 maize varieties, we selected the salt-sensitive variety Yunyu7 (YY7) and the salt-tolerant variety Jingnongyu658 (JNY658) as the experimental subjects. The elite Chinese maize cultivar Jingnongyu658 (JB547/J2418) and Yunyu 7 (14NC7 × 15S856) were obtained from Shandong Jingke Seeds Co., Ltd. (Jinan, China) and Yuncheng Seeds Co., Ltd. (Heze, China), respectively. The study complies with relevant institutional, national, and international guidelines and legislation for plant ethics. The factors were two salt levels: 0 mM NaCl (CK) and 100 mM NaCl (S), and two varieties (YY7, JNY658).

### Experimental design of the mesocosm experiment

The experiment was divided into two branches. One branch used a soil environment to grow the experimental plants and the other a hydroponic environment.

The mesocosm experiment was carried out in a greenhouse, using a 2 × 2 factorial randomized complete block design blocked by salinity treatment and maize variety. Seeds of the same size and fullness were surface-sterilized for 10 min with a 0.05% NaClO solution, washed for 30 min with distilled water, and then soaked for 24 h in distilled water. Plants were grown in soil containers measuring 35 × 35 × 40 cm (length × width × height). Each treatment per variety was replicated over 18 plants, for a total of 72 plants. The containers were filled with a mixture of the substrate we prepared and soil in a 4:1 ratio (V: V), and placed in a greenhouse with a relative humidity of 70% and a photoperiod of 16 hours of light and 8 hours of darkness. Growth conditions consisted of 1200 μmol photons m^− 2^ s^− 1^ maximum photosynthetically active radiation. After the seeds had finished soaking, the containers were planted with three seeds each. Approximately 10 days after seeding, they were thinned to one plant per container. During the growing period, the plants were provided with enough water and mineral nutrients to meet their requirements, as calculated using the formula proposed by Kalaji et al. [85]. Up to the three leaves development stage, each container was irrigated three times per week with 1000 mL of half-strength Hoagland’s culture solution. After reaching the third fully expanded leaf stage (V3), the plants were irrigated with 1000 ml of standard Hoagland nutrient solution laced with 100 mM NaCl three times per week for salt stress treatment (S), and the control treatment (CK) was irrigated using 1000 ml of standard Hoagland nutrient without added NaCl three times per week.

### Determination of the morphological traits of shoots

Six plants per treatment were selected, and their leaf length (L) and maximum leaf width (W) were measured. The measurements were then used to calculate the leaf area, using the following formula: leaf area (cm^2^) = leaf length (cm) × leaf width (cm, the widest part of the leaf) × 0.75.

### Determination of the RWC

To determine the RWC, we collected whole fresh leaves and immediately measured the fresh leaf width (Wf). The collected leaves were immersed for 6–8 h in distilled water, after which they were removed from the bath and water drops left behind on the surface were wiped off with absorbent paper. After this, the leaves were weighed. To obtain the saturated fresh leaf weight (Wt), we soaked the samples for another 1 h in distilled water, removed them from the bath, wiped off any water drops and weighed them again. Finally, we dried the leaves to determine the dry weight (Wd). RWC was calculated as: RWC = (Wf − Wd)/ (Wt − Wd) × 100%.

### Measurement of photosynthetic characteristics

At V6 stage, the Pn, Tr, Gs, and Ci in the youngest fully expanded leaf of each plant were measured with a CIRAS-III portable photosynthesis system (PP System, Hansatech, UK) using an LED light with a PAR intensity of 1600 μmol photons m^− 2^ s^− 1^. The temperature and the CO_2_ concentration in the leaf chamber were kept at 25 °C and 390 μmol mol^− 1^, respectively.

### Measurement of the antioxidant enzyme activity and the MDA content

To measure the enzyme activity in plants from the soil culture experiment, we collected leaf and root samples of six plants for each treatment freeze-dried them in liquid nitrogen, and fully ground them to powder. Then, we took 0.5 g samples, added 5 ml phosphoric acid buffer (pH = 7.8), centrifugated them for 20 min, placed the supernatant into test tubes and stored them at 0–4 °C until analysis.

The SOD activity was determined according to the method proposed by Wang [86]. We prepared a SOD reaction solution consisting of 1.5 ml pH 7.8 phosphoric acid buffer, 0.3 ml methionine (130 mM), 0.3 ml tetrazolium blue (750 μM), 0.3 ml EDTA-Na_2_ (100 μM), 0.3 ml riboflavin (20 μM) and 0.3 ml distilled water. Using a pipette, we added 20 μl of sample to 3 ml of the reaction solution. Some of the samples were illuminated for 30 minutes at 4000 lx, and some, representing the blank treatment, were placed in darkness. To measure the SOD activity, the samples were analyzed using UV–visible spectrophotometer at 560 nm.

The POD activity was determined according to the method proposed by Giannopolitis [87]. We prepared a POD reaction solution consisting of 50 ml phosphoric acid buffer containing 0.1 mM pH 6.0, 28 μl guaiacol, and 19 μl 30% H_2_O_2_. After cooling the reaction solution, we used a pipette to add 20 μl of samples to 3 ml of the reaction solution. Using a UV–visible spectrophotometer, we measured the rate at which the absorbance at 470 nm changed, taking one measurement per min for a total of 3 measurements. The average rate at which the absorbance changed was used to represent the enzyme activity.

The CAT activity was determined according to the method proposed by Reis et al. [88]. We prepared a CAT reaction solution consisting of 5 ml of 0.1 mM H_2_O_2_ and 20 ml of phosphoric acid buffer at a pH of 7.0. Using a pipette, we added 50 μl of sample to 2.5 ml of the reaction solution. Using a UV–visible spectrophotometer, we measured the rate at which the absorbance at 240 nm changed, taking one measurement per min for a total of 3 measurements. The average rate at which the absorbance changed was used to represent the enzyme activity.

The MDA content was determined using the thiobarbituric acid method described by Lin [89]. 1 ml of sample was added to a reaction solution consisting of 2 ml of 0.6% thiobarbituric acid. The test tube holding the resulting solution was immersed in a bath of boiling water, then cooled and finally centrifuged. The MDA content was determined by measuring the absorbance of the supernatant at 600 nm, 532 nm and 450 nm, using UV–visible spectrophotometer.

### Characterization of the root anatomy

To analyze the root anatomy, we collected the whole plant root system of six plants per treatment from the mesocosm experiment. We manually excised a 4 cm root segment from each plant at 5 cm from the base of the whorl of crown roots and stored it in a 75% alcohol solution. The roots were sliced by hand, dying them with safranin, and observing them under a DM21-J1200 optical microscope. The cross sections were photographed with Scope Image 9.0 software, and the resulting pictures were used to characterize various root anatomy features with the RootScan Structure software [90].

### Measurement of the dry matter weight and calculation of the salt tolerance index

At V6 stage, we harvested six plants per treatment by dividing them into roots and shoots, placing them in an oven, drying them at 80 °C to constant dry weight, and finally weighing them.

The salt tolerance index (STI) [16] was calculated according to the following formula:

$$STI\ \left(\%\right)=\frac{Total\ dry\ weight\ of\ ssedlings\ subjected\ to\ salt\ stress}{Total\ dry\ weight\ of\ seedling\ from\ the\ control\ group}\times 100$$

### Measurement of the Na^+^ content, uptake and translocation

To measure the Na^+^ content of the harvested seedlings, we milled the dried samples used to measure the dry weight and digested them for 4 h with an H_2_SO_4_-H_2_O_2_ solution at 340 °C. Subsequently, we measured the Na^+^ content with an F-410 Flame Photometer (Cambridge). The Na^+^ uptake through the maize root surface and the Na^+^ translocation from roots to shoots were calculated using the following equations proposed by Shahzad [91].

$${Na}^{+} uptake\ through\ root\ surface=\frac{Total\ {Na}^{+} content}{Root\ dry\ weight}$$

$$Translocation\ factor=\frac{Na^{+}\ concentration\ in\ leaves}{Na^{+}\ concentration\ in\ roots}$$

### Experimental design of the hydroponics culture experiment

The hydroponic culture experiment was carried out in a light- and temperature-controlled room at Shandong Agricultural University in Tai’an, Shandong, China. We selected 50 seeds of the same size and sterilized them by soaking them for 15 min in a 0.05% NaClO solution. We left the seeds for 2 days in the dark on a sheet of 0.5 mM CaSO_4_ germination paper, after which we selected seeds that had germinated at the same growth rate and transferred them to a hydroponic tank with a length, width and height of 55 cm × 42 cm × 23 cm. After the seedlings were left to grow until they reached the third fully expanded leaf (V3) stage, the S treatment was soaked into Hoagland nutrient solution with a 100 mM NaCl solution three times per week, while those that were to receive the CK treatment were soaked in a modified Hoagland nutrient solution without added NaCl. During the entire growth period, all plants were provided with amounts of water and mineral nutrients that met their requirements, as calculated using the formula proposed by Kalaji et al. [85], and we installed an air pump to ensure sufficient oxygenation of the nutrient solution. Plants were grown at a temperature of 28 °C during the day and 25 °C during the night, with a photoperiod of 16 hours of light and 8 hours of darkness.

### Measurement of the net Na^+^ and K^+^ flux

The seedlings treated with 0 and 100 mM NaCl for 24 h were used to measure the net Na^+^ and K^+^ fluxes. To measure the net Na^+^ and Ka^+^ flux, we collected 2–3 cm segments from the apexes of roots from the third layer of nodal shaft roots of six plants from each treatment group. They were rinsed with redistilled water and immediately immersed in measurement solution where they were left to equilibrate for 30 min. After that, the samples were transferred to the measuring chamber of a non-invasive micro-measuring system (NMT; Younger, USA) containing 10–15 ml of fresh measurement solution. At the measurement site, which was located inside the elongation zone of the root at 400 μm from the root apex, we typically observed a vigorous K^+^ and Na^+^ flux. For Na^+^ flow rate measurements, we used a measurement solution consisting of 0.1 mM KCl, 0.1 mM CaCl_2_, 0.1 mM MgCl_2_, 0.5 mM NaCl, 0.3 mM MES, 0.2 mM Na_2_SO_4_, and for K^+^ flow rate measurements, it consisted of 0.1 mM KCl, 0.1 mM CaCl_2_, 0.1 mM MgCl_2_, 0.3 mM MES, 0.2 mM Na_2_SO_4_. The NMT measurement results were used to calculate the net ion fluxes using the JCal V3.3 software (xuyue.net).

### Measurement of the root respiration rate

At V6 stage, we measured the total root respiration rate and the alternate oxidation respiration rate, the roots of six plants for each treatment were sampled, and the 8–18 cm segments were excised from the axial and lateral roots at the base of the third root layer from plants. To avoid wound respiration, we left the root segment samples to rest on moist cotton gauze for 5 minutes. After that, we placed them in the liquid chamber of an Oxytherm+R oxygen electrode system (Hansatech, UK), and added 2 ml of the nutrient solution used in the treatment of the group from which the plant under study was taken (pH = 6.0 ± 0.1). Next, we measured the total respiration rate of the root sample over 20 min in the dark and calculated the R_total_ from the slope of the curve describing the change in oxygen concentration as measured from 5 to 20 min. Subsequently, we added 2 ml 25 mM SHAM solution into the liquid chamber, which allowed us to measure R_SHAM_ due to the fact that adding SHAM inhibits the R_AOX_. Finally, we used the measured values of R_total_ and R_SHAM_ to calculate R_AOX_ according to the following formula: R_AOX=_ R_total_- R_SHAM_.

### Data analysis

The effects of and the interactions between the two main experimental factors (the maize variety and exposure to salt stress) were analyzed through a two-way analysis of variance (ANOVA) using the SPSS 17 software (er.17.0, SPSS, Chicago, IL, United States). Tukey’s test was used to compare multiple treatments. Correlation analysis (*P* < 0.05) between various measured attributes of maize varieties. Correlation analysis (*P* < 0.05) between various measured attributes of maize varieties was performed by Origin2021 (OriginLab, Northampton, Massachusetts, USA). The figures were constructed using the SigmaPlot 12.5 software (Systat Software, Inc., San Jose, CA).

## Data Availability

The datasets used and analyzed during the current study are available from the corresponding author on reasonable request.
